# Accelerated Genetic Gains in Early-Maturing Maize Hybrids following Three Periods of Genetic Enhancement for Grain Yield under Low and High Soil-Nitrogen Environments

**DOI:** 10.3390/plants11091208

**Published:** 2022-04-29

**Authors:** Baffour Badu-Apraku, Morakinyo Abiodun Bamidele Fakorede, Adamu Masari Abubakar

**Affiliations:** 1 International Institute of Tropical Agriculture (IITA), PMB 5320, Ibadan 200001, Nigeria; a.abubakar@cgiar.org; 2 Department of Crop Production and Protection, Obafemi Awolowo University, Ile-Ife 220282, Nigeria; delefakoredemab@gmail.com

**Keywords:** breeding period, GGE biplot, hybrids, genetic gains, low N tolerance

## Abstract

Maize (*Zea mays* L.) is an important staple, as well as cash crop, in sub-Saharan Africa (SSA). However, its production is severely constrained by low soil nitrogen (low N). Fifty-four early-maturing hybrids developed during three breeding periods, (2008–2010, 2011–2013 and 2014–2016) were evaluated under low (30 kg ha^−1^) and high (120 kg ha^−1^) soil nitrogen (N) in Ile-Ife and Mokwa, Nigeria, from 2017 to 2019. The study was designed to (i) determine the genetic gains in grain yield of the early-maturing maize hybrids developed during the three breeding periods, (ii) determine the relationship between grain yield and other agronomic traits and (iii) identify the highest-yielding and most stable hybrids under low- and high-N environments. The 54 hybrids were evaluated using a 6 × 9 lattice design with three replications. Mean squares for hybrids were significant for measured traits under low- and high-N environments, except the mean squares for stalk lodging and EPP under low N. Annual genetic gains in grain yield were 75 kg ha^−1^ year^−1^ (2.91%) and 55 kg ha^−1^ year^−1^ (1.33%) under low- and high-N environments, respectively, indicating that substantial gains were achieved in the genetic enhancement of the early-maturing hybrids. The hybrids TZdEI 314 × TZdEI 105, TZdEI 378 × TZdEI 173, ENT 12 × TZEI 48 and TZdEI 352 × TZdEI 315 were identified as the highest-yielding and most stable across test environments and should be tested extensively on farms and commercialized in SSA.

## 1. Introduction

Maize (*Zea mays* L.) is one of the most important staple foods and cash crops in SSA. It has the potential to mitigate the present food insecurity in the sub-region. Annual maize production in West and Central Africa (WCA) is estimated at about 11 million metric tons [1]. It is produced largely in the savannah zones and, to a lesser extent, in the forest and mid-altitude agro-ecologies, which are commonly affected by typical biotic and abiotic stresses, prominent among which are inherent poor soil fertility, recurrent drought, diseases and pests, including armyworm. In addition, the savannah ecologies are typically infested by *Striga harmonthica* [2,3].

The International Institute of Tropical Agriculture (IITA) initiated a maize research program in the 1970s targeted to stress environments in SSA. The program in collaboration with the National Agricultural Systems (NARS) of the sub-region and the West Africa Collaborative Maize Research Network (WECAMAN) has focused on breeding materials with resistance to the major diseases, namely downy mildew, maize streak virus, rust and leaf blight for all agro-ecologies; borer resistance for the forest agro-ecology; and resistance/tolerance to *Striga*, drought and low nitrogen use efficiency for the savannah agro-ecology. Under field conditions, drought, *Striga* and soil nutrient deficiencies can occur simultaneously, and the combined effect can be devastating [4,5]. Some early-maturing *Striga*-resistant, drought-tolerant inbred, open-pollinated and hybrid cultivars were developed early in the program by the International Institute for Tropical Agriculture-Maize Improvement Unit (IITA-MIP); such cultivars were also found to be low N-tolerant, although they were not deliberately selected for tolerance to low N until 2000. Low N stress in SSA is caused by several factors, including the widespread removal of crop residues for use as animal feed and fuel and leaching of soil N below the root zone due to heavy rainfall [6]. Additionally, poor weed control in farmers’ fields increases the incidence of N deficiency, which is worsened by the application of sub-optimal levels of inorganic fertilizer due to high prices and the unavailability of fertilizer [7]. Nitrogen is the most limiting nutrient in tropical soils. Consequently, lack of or low N availability occurring early in the life of the plant reduces plant leaf area expansion, as well as photosynthesis [6]. If nutritional stress occurs just before flowering, it causes severe damage when structures that determine yield are formed. The low level of nitrogen in tropical soils is a constraint to high productivity if fertilizers, either organic or inorganic, are not adequately applied. Available nitrogen is reduced by losses through running water, volatilization, leaching and nitrogen taken up by plants. Estimated yield losses due to N stress alone range between 1 and 50% [8]. Therefore, to alleviate the effects of low N stress, the IITA-MIP has embarked on the development of low N tolerant genotypes over the last three decades, resulting in the development of several low N-tolerant populations, inbred lines and hybrids. The availability of early-maturing maize in the savannas of SSA has boosted maize production and consumption either as green maize or dry grain and has helped to fill the hunger period in July in the savannas of the sub-region, during which other food reserves are depleted due to the long dry period [9]. Additionally, the availability of early-maturing maize hybrids has resulted in considerable increases in maize productivity and production, leading to remarkable improvements in the incomes and well-being of farmers in the sub-region. However, low soil nitrogen, drought and *Striga hermonthica* parasitism still constitute major limitations to maize production and productivity in SSA. Therefore, to mitigate the effects of low N stress, the IITA-MIP has embarked on the development of low N tolerant, open-pollinated, early-maturing cultivars and hybrids that can utilize available nitrogen more efficiently. Germplasms with good nitrogen use efficiency were identified following several years of extensive testing in SSA and have been used as parents to develop adapted source populations for improvement using recurrent selection methods. Several promising early-maturing varieties with high yield under low nitrogen have been identified and commercialized in SSA.

Several authors [10,11,12,13,14,15] have reported genetic gain studies for comparisons of older and newer cultivars under different environments, and they have significantly contributed to better understanding of the breeding progress of maize cultivars developed during different periods [16,17]. Results of genetic gain studies have revealed significant increases in grain yield between generations of cultivars. For example, the authors of [9,15,18] reported genetic gain in grain yield of 1.7% per era in the evaluation of 50 early-maturing cultivars under *Striga*-infested conditions and 1.1% under drought stress. A genetic gain of 1.3% year^−1^ was reported, with an increase in grain yield of 30 kg ha ^−1^, under low and high N. Additionally, Badu-Apraku et al. [15] indicated annual genetic gains of 44 kg ha^−1^ year^−1^ and 2.72% for 56 extra-early OPVs under contrasting stress environments (drought, low N and *Striga* infestation). Similarly, Badu-Apraku et al. [18] reported 2.56% annual gains from selection of extra-early OPVs, with a mean increase in rate of gain yield of 42 kg ha^−1^ year^−1^ under *Striga*-infested conditions.

To improve maize production and productivity under low N conditions, indirect selection is vital [19,20,21], as this strategy increases gains from selection for grain yield through the exploitation of specific adaptation. Furthermore, the development of high-yielding and stable maize hybrids with tolerance to low N is important for increased maize productivity, and this offers the most economic and sustainable approach for increased maize yields by small-scale farmers who utilize low N inputs in SSA. Breeding of early-maturing maize for tolerance to low N in IITA-MIP has covered three specific periods from 2008–2016. However, no study has been reported on the accelerated gain in breeding progress of the early-maturing hybrids generated during the last three periods, thus making it difficult to completely ascertain the genetic gain that has been made in grain yield in relationship to N fertility in the numerous early-maturing hybrids that have been developed and released in SSA. Therefore, the objectives of the present study were to: (i) evaluate, under low and high soil N conditions, the genetic gains in grain yield of early-maturing maize hybrids developed during the three breeding periods (2008–2010, 2011–2013 and 2014–2016), (ii) determine the relationship between grain yield and other agronomic traits of early-maturing maize hybrids and (iii) identify high-yielding and stable hybrids under low and high N environments for commercialization in SSA.

## 2. Results

### 2.1. Analyses of Variance and Genotype by Environment Interaction of Grain Yield and Other Agronomic Traits

Results of the combined analysis of variance (ANOVA) across the four test environments under low N and five test environments under high N showed significant (*p* < 0.05) mean squares for environment (E), period and hybrid (period) for grain yield and other measured traits, except in a few cases in which days to anthesis, days to silk, anthesis silking interval (ASI), root and stalk lodging and ear rot for period were not significant (Table 1). Additionally, significant (*p* < 0.05) mean squares for E × hybrid (period) and E × period interactions were observed for grain yield and most measured traits, except for plant height, ear height, root and stalk lodging for E × period interaction under high N. Similarly, mean squares for ear height, root and stalk lodging, ears per plant (EPP) and stay-green characteristic were not significant under low N for the E × hybrid (period) interaction.

### 2.2. Repeatability Estimates of Grain Yield and Other Agronomic Traits

Repeatability estimates of the measured traits across the test environments ranged from 0.14 for root lodging to 0.84 for days to anthesis under low N and 0.24 for EPP to 0.86 for plant height under high N. (Table 1). In general, moderately high repeatability estimates (i.e., ≥0.30) were recorded for measured traits across low N environments, except for EPP and root and stalk lodging, which were less than 0.30. Similarly, in high N environments, moderately high repeatability estimates were recorded for the measured traits, except for EPP. Under low and high N, days to anthesis had the highest repeatability estimates of 84 and 86%, whereas estimates for grain yield were 56 and 59% under low and high N, respectively.

### 2.3. Genetic Improvements in Grain Yield and Other Measured Traits

**Figure 1:**
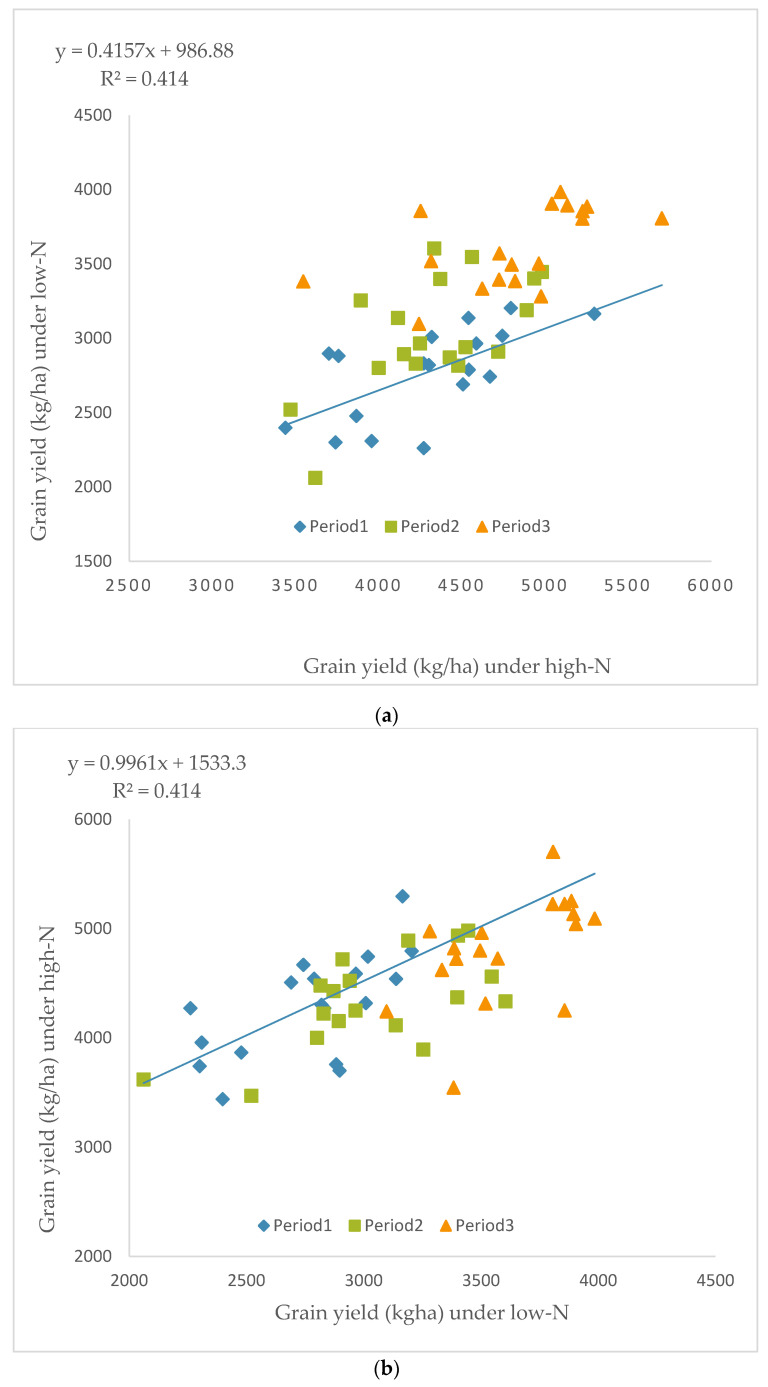
Comparative performance of early-maturing maize hybrids of the three breeding periods in low and high N environments. Regression of (**a**) grain yield of low N on grain yield of high N and (**b**) grain yield of high N on grain yield of low N conditions.

A significant (*p* < 0.05) increase in grain yield was obtained during the third period (period 3) of the early maize hybrid cultivation compared to those developed during the first and second periods in low and high N environments (Table 2). Under low N, grain yield increased from 2784 kg ha^−1^ during the first period to 2933 and 3197kg ha^−1^ during the second and third periods, respectively, with a relative genetic gain of 2.91% per year and an average rate of increase in grain yield of 75.37 kg ha^−1^ year^−1^ (Table 2 and Table 3). Similarly, under high N environments, grain yield improved from 4294 kg ha^−1^ during the first period to 4330 kg ha^−1^ during the second period and to 4614 kg ha^−1^ during the third period, although grain yield in period 2 was not significantly different from that of period 1. Under high N, there was genetic gain of 1.33% per year and an average rate of increase of 55.12 kg ha^−1^ year^−1^ (Table 3). The significant gain from selection for grain yield between periods 1 and 3 observed under low N and high N environments was associated with reduced ASI, as well as improved stalk lodging, husk cover, stay-green characteristic, plant and ear aspects. Additionally, significant positive b values were observed for yield, plant height and EPP. In contrast, significant negative b values were recorded for plant and ear aspects under low- and high N conditions and ASI under low N environments (Table 3). Regression analysis of the yield of the maize hybrids under low N on yield under high N (Figure 1a) and yield under high N on yield under low N environments clearly differentiated period 3 hybrids from period 1 and 2 hybrids. Despite the overlaps in performance of the hybrids of the three periods, grain yield of period 3 hybrids was the best in both research environments. Similarly, regression analysis of grain yield under high N environments showed a positive predictive relationship between one level and the other, with performance under low N predicting performance under high N.

### 2.4. Correlation of Grain Yield and Other Agronomic Traits

Results of Pearson’s correlation analysis revealed significant positive correlations between grain yield and plant height (r = 0.47 **), ear height (r = 0.26 *) and EPP (r = 0.49 **). Contrarily, significant negative correlations were observed between grain yield and plant aspect (r = −0.65 **), ear aspect (r = −0.61 **) and stay-green characteristic (r = −0.29 **) in low N environments (Table 4). In high N environments, grain yield had significant and positive correlations with plant height (r = 0.32 **) and EPP (r = 0.60 **), whereas significant and negative correlations were detected between grain yield and ASI (r = −0.38 **), plant aspect (r = −0.59 **) and ear aspect (r = −0.80 **). Additionally, plant height had significant positive correlations with days to anthesis (r = 0.27 **; 0.36 **) and days to silking (r = 0.27 **; 0.33 **) but had significant negative correlations with plant aspect (r = −0.59 **; −0.55 **) under high and low N, respectively.

### 2.5. Grain Yield Performance and Stability of Maize Hybrid in Low- and High N Environments

**Figure 2:**
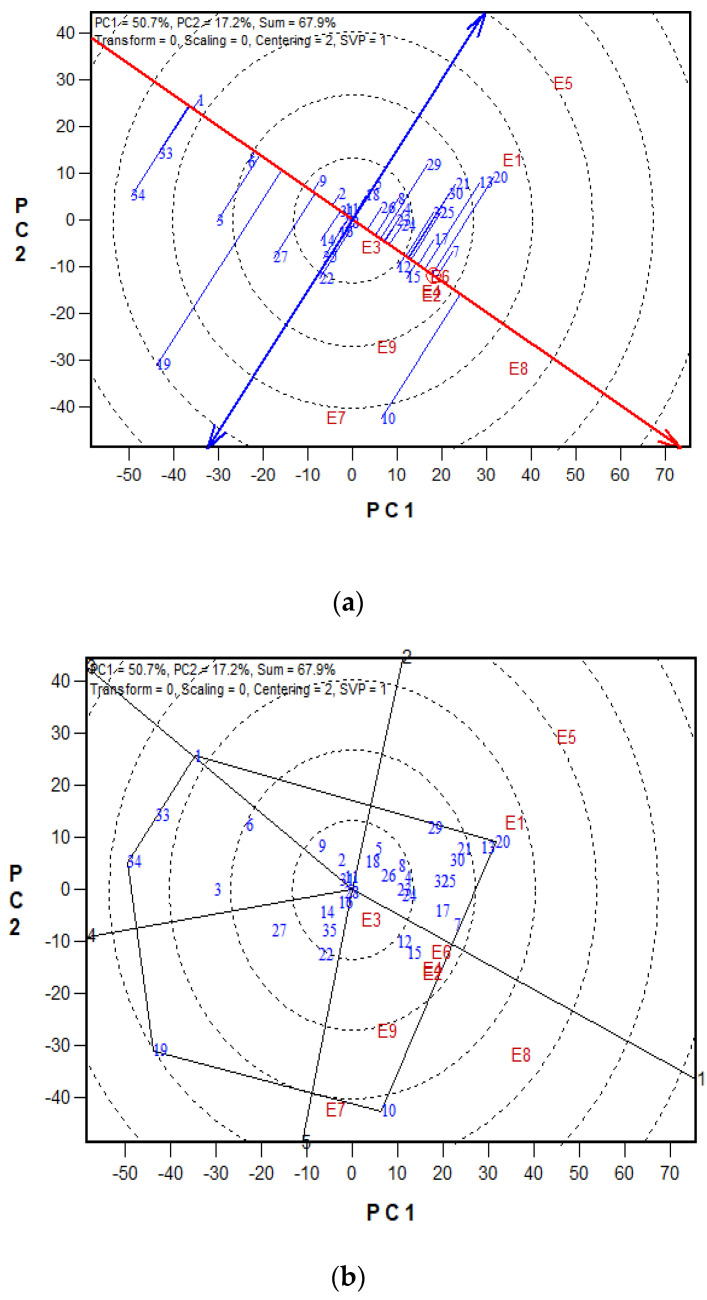
(**a**). The “mean vs. stability” view of the genotype main effect plus genotype × environment interaction (GGE) biplot based on genotype × environment yield data of 35 early-maturing maize hybrids evaluated in five low N environments and four high N environments in Nigeria from 2017 to 2019. E1 = Ile−Ife low N 2017; E2 = Ile−Ife low N 2018; E3 = Mokwa low N 2018; E4 = Ile−Ife low N 2019; E5 = Ile−Ife high N 2017; E6 = Mokwa high N 2017; E7 = Mokwa low N 2017; E8 = Ile−Ife high N 2018; E9 = Mokwa high N 2018. (**b**). A ‘which-won-where’ GGE biplot of grain yield of 35 early-maturing maize hybrids evaluated across five low N and four high N environments in Nigeria from 2017 to 2019. E1 = Ile−Ife low N 2017; E2 = Ile−Ife low N 2018; E3 = Mokwa low N 2018; E4 = Ile−Ife low N 2019; E5 = Ile−Ife high N 2017; E6 = Mokwa high N 2017; E7 = Mokwa low N 2017; E8 = Ile−Ife high N 2018; E9 = Mokwa high N 2018.

The GGE biplot of the 35 (best 15, middle 10 and worst 10) early-maturing hybrids revealed that PC1 explained 50.7% of the total variance, whereas PC2 explained 17.2%, together accounting for 67.9% of the total variation among the hybrids evaluated across test environments. In the average-tester-coordination view of the GGE biplot (Figure 2a), the absolute length of the projection of the hybrid to the ATC (double-arrow blue horizontal lines) is a measure of its stability, whereas the single-arrow line (*x*-axis) is the average environment coordination (AEC) axis and points in the direction of higher mean grain yield. The shorter the projection, the more stable the hybrid. Therefore, the hybrids TZdEI 314 × TZdEI 105, TZdEI 378 × TZdEI 173, ENT 12 × TZEI 48 and TZdEI 352 × TZdEI 315 were the most stable across test environments and had considerably high yield performance, which was far above average (Figure 2a). The hybrid TZdEI 173 × TZdEI 280 had the highest yield performance across environments, whereas TZE-Y Pop DT C5 STR C5 × TZEI 17 had the lowest grain yield. TZdEI 479 × TZdEI 124 and TZdEI 173 × TZdEI 280 were the least stable hybrids across test environments. According to Yan et al. (33) an ideal genotype should have a high mean grain yield and high stability. Thus, hybrids TZdEI 314 × TZdEI 105, TZdEI 378 × TZdEI 173, ENT 12 × TZEI 48 and TZdEI 352 × TZdEI 315 were identified as ideal hybrids. The which-won-where view of the biplot separated the environments into two mega environments (Figure 2b). Hybrids 20 (TZdEI 17 × TZEI 17), 13 (TZdEI 173 × TZdEI 492), 21 (TZdEI 68 × TZEI 10) and 29 (TZE-W Pop DT C5 STR C5 × TZEI 63) performed best at Ife low and high N (2017). Similarly, hybrids 10 (TZdEI 173 × TZdEI 280), 12 (TZdEI 314 × TZdEI 105) and 15 (TZdEI 378 × TZdEI 173) performed best in all other environments.

## 3. Discussion

Significant mean squares detected for grain yield and other measured traits in both low- and high N environments indicate that the test environments were unique in identifying superior hybrids. The presence of significant differences among the hybrids for grain yield and most measured traits in both low- and high N environments indicates the existence of genetic variability among the early-maturing hybrids developed by IITA-MIP during the three periods of genetic enhancement. The existence of high genetic variability among the hybrids is expected to facilitate accelerated genetic gains from selection for improvements in grain yield and other studied traits. This is also expected to facilitate identification of sources of genetic variability for development of new inbred lines, synthetics and populations with superior genes.

Differential response of genotypes to varying environmental conditions constitutes a major challenge in the identification of superior maize hybrids for wide or narrow adaptation [17]. The significant hybrid × environment interactions observed for grain yield and most measured traits in low and high N environments indicate that the expression of these traits would not be consistent in contrasting test environments. This result suggests the need for extensive testing of the hybrids in multiple environments under different conditions for identification of genotypes with consistent performance under varying environmental conditions. These results are consistent with the findings of Badu-Apraku and Oyekunle [22]. Contrarily, the lack of significant hybrid × environment interaction observed for the stay-green characteristic and EPP under low N and for ear height, root and stalk lodging under low and high N indicates that the traits were not affected by hybrid × environment interactions and hence the expression of these traits would be consistent in varying test environments. The high repeatability estimates (≥60%) observed in the present study for most measured traits under low and high N conditions suggests that the expression of these traits would be consistent in varying environments.

In the present study, the genetic gain of 2.91% year ^−1^ with the average rate of increase in grain yield of 75.37 kg ha ^−1^ year ^−1^ obtained for the early-maturing hybrids under low N stress and the annual genetic gain of 1.33% under high N, with an average increase of 55.22 kg ha^−1^ year^−1^, is higher than the gains reported for the early-maturing, open-pollinated cultivars [15] under low and high N conditions. The authors reported an average rate of increase in grain yield of 30 kg ha ^−1^ year ^−1^, with r^2^ values of 0.99 and 0.94 under low and high N conditions, respectively. Additionally, the obtained genetic gains were greater than the 2.14% under low N and the 2.56% under *Striga* infestation documented by Badu-Apraku et al. [17]. The percent gain per year obtained in the present study is also greater than the 1.93% per year documented in [21] and [14] when 24 open-pollinated, extra-early and 23 commercial maize varieties were assessed under drought conditions in Kadawa and Zaria, Nigeria, in the 2015/2016 and 2016/2017 dry seasons. The implication of these findings is that hybrids respond favorably to selection compared to open-pollinated varieties. It is therefore of interest that IITA-MIP focus extensively on development of hybrids rather than open-pollinated varieties. Contrarily, the percent gain of 2.91% year^−1^ with an average rate of increase in grain yield of 75.37 kg ha ^−1^ year^−1^ obtained under low N in the present study is lower than the annual increase in grain yield of 101 kg ha^−1^ and gain of 4.82% year^−1^ recorded when the same early-maturing hybrids as those used in the present study were evaluated under *Striga* infestation [23]. In a similar study involving the same early-maturing hybrids of the three breeding periods evaluated under drought stress, a genetic gain of 4.14% year^−1^ was obtained [unpublished]. In the three different studies with the same early-maturing hybrids derived from the three breeding periods, genetic gains of 1.43, 1.24 and 1.33% year^−1^, with average increases of 61.1, 61.0 and 55.1 kg ha^−1^ year^−1^ were obtained under optimal (non-Striga infested), well-watered and high N conditions, respectively. The implications of these results are that early-maturing hybrids responded better to selection for improved *Striga* resistance, as well as drought, compared to the low N and optimal conditions. This result is not surprising because during the beginning phase of the early and extra-early maize program, a major interest of the IITA–MIP was to select maize inbred lines with enhanced adaptation to drought-prone environments from diverse sources. The promising selected inbred lines were then screened for *Striga* resistance under artificial *Striga* infestation. The inbreds with better adaptation to both drought and/or genes for drought tolerance during the flowering and grain-filling periods, as well as *Striga* resistance genes from *Zea diploperennis*, were used as sources of genes for further introgression into the early-maturing breeding populations. Additionally, the smaller gains from selection for grain yield of the early-maturing hybrids under high N conditions affirmed that IITA-MIP maize breeders had been focused on improving stress tolerance rather than enhanced performance of the hybrids in optimal environments. In the present study, the significant gain in grain yield in low N environments was associated with reduced ASI and improved stalk lodging, husk cover and stay-green characteristic, as well as plant and ear aspects. This suggests that the selection index used by the IITA-MIP for improving yield under low N resulted in delayed flowering; reduced ASI; improved husk cover, plant and ear aspects; as well as improved stalk lodging [24]. In fact, a substantial increase in the grain yield of the early-maturing maize hybrids was achieved under low N conditions during the three breeding periods of genetic enhancement. Regression of grain yield of hybrids (periods 1 to 3) under low N on the grain yield under high N conditions clearly demonstrated the high grain-yielding ability of the period 3 hybrids compared to those of periods 1 and 2. This confirmed the magnitude of the progress made in enhancing the grain yield of the period 3 hybrids. Results of the regression analysis confirmed the superiority of period 3 hybrids under both low and high N conditions compared to those of periods 1 and 2. These results therefore justify the efforts and resources devoted by the IITA-MIP to increase genetic gains through selection of early-maturing maize hybrids.

Information on the relationships among traits is important for designing effective strategies in breeding programs for maize improvement [14]. The significant correlations recorded between grain yield and EPP, plant and ear height, ASI, plant and ear aspects under both low and high N conditions indicate that those traits could serve as important secondary traits for improvement of grain yield under both low and high N conditions. The presence of significant correlations between grain yield and plant and ear heights indicates that taller hybrids tend to give higher yields than shorter hybrids. Contrarily, significant correlations between grain yield and EPP, ASI, and plant and ear aspects indicate the possibility of using these secondary traits in improvement of grain yield, thus justifying the inclusion of the traits in the selection index for the identification and improvement of low N tolerant genotypes. These results are consistent with the findings reported in [22,25]. However, although the lack of significant correlations between grain yield and days to 50% anthesis and silking under both low and high N conditions are in agreement with the findings of previous researchers [17,26], the results are in disagreement with the findings reported in [14]. The presence of significant correlations between pairs of traits, such as plant and ear heights, days to anthesis and silking, and plant and ear aspects under both low and high N conditions (Table 4) indicates that improving one among a pair of traits could lead to improvements in the other. This is important because doing so could reduce the cost of measuring two different traits that provide similar information [14].

The GGE biplot is an invaluable means for the identification of ideal genotypes across multiple environmental conditions. The hybrids TZdEI 314 × TZdEI 105, TZdEI 378 × TZdEI 173, ENT 12 × TZEI 48 and TZdEI 352 × TZdEI 315 were identified as high-yielding and stable under both low and high N conditions (Figure 2a), suggesting that they had broad adaptation to the growing environments in SSA. The results of the present study are of particular interest because, except hybrid ENT 12 × TZEI 48, all the outstanding hybrids identified in the present study were from period 3. The results clearly indicate that the hybrids developed during period 3 possess beneficial alleles that contributed to the observed superior performance compared to the hybrids developed during periods 1 and 2. In the which-won-where view (Figure 2b), the axes, starting from the origin, separate the biplot into sectors or mega environments, with the hybrids at the vertices of each sector representing the best-performing hybrid(s) within a given sector. This facilitated the process of identifying the superior hybrids for specific environments [27]. From the polygon view, TZdEI 17 × TZEI 17, TZdEI 173 × TZdEI 492 and TZdEI 68 × TZEI 10 were identifies the best hybrids in Ile-Ife under low and high N, whereas TZdEI 173 × TZdEI 280 was the best in Mokwa under low N.

## 4. Materials and Methods

### 4.1. Development of Low NTolerant, Early-Maturing Inbreds and Hybrids for the Present Study

The early-inbred line development program was initiated in 1994 under the West and Central Africa Maize Collaborative Research Network/International Institute of Tropical Agriculture (WECAMAN/IITA). It began with several broad-based germplasm sources with resistance to Striga and MSV, as well as tolerance to drought, including TZE-W Pop DT STR C0, TZE-Y Pop DT STR C0, TZE Comp 5-Y C6 and TZE-W Pop × 1368 STR. The selected S1 lines extracted from each population were evaluated under Striga-free conditions in Sinematialli and Ferkessedougou under artificial infestation. The combining abilities for grain yield, Striga damage rating, number of emerged Striga plants, number of ears per plant, yield performance of the lines per se and other desirable agronomic characters across the locations were adopted as criteria for selecting between 90 and 100 S4 lines and advancing to the S6 stage of inbreeding. The breeding strategies adopted in developing the inbreds and hybrids utilized in the present study are described in detail in [3,17]. A total of 54 hybrids, 18 each of periods 1, 2 and 3, were identified as possessing superior performance in the regional trials for the respective breeding periods.

### 4.2. Field Evaluation

A total of 54 hybrids representing three breeding periods (2008–2010, 2011–2013 and 2014–2016) were selected for the present study (Supplementary Table S1). The selection of the hybrids was based on superior performance in the regional trials within SSA, with several of them having the same female parent, independent of year of release. The trials were evaluated under low and high N conditions following the depletion of N at each experimental site. The trials were conducted at the Teaching and Research Farm, Obafemi Awolowo University, Ile-Ife (4°32′ E, 7°28′ N, 280 m above sea level, 1300 mm average rainfall yearly) in the forest–savannah transition and Mokwa (latitude 9°18′ N, longitude 5°4′ E, 457 m above sea level, 1100 mm yearly average rainfall) in the southern Guinea savanna of Nigeria during the 2017, 2018 and 2019 growing seasons. The soil in Ile-Ife is characterized as Alfisol [28], with 0.084% organic N, whereas the soil in Mokwa is a Luvisol [28], with 0.27, 0.035 and 0.48% organic C, organic N and P content, respectively. N depletion was achieved by continuously planting maize over years and removing stover from the field after each harvest. Soil samples were taken each year before planting in all the test environments, and N content was determined at the IITA soil laboratory in Ibadan. The total N in the soils was determined by Kjeldahl digestion, and colorimetric determination was performed on a Technicon AAII Auto analyzer. A 6 × 9 incomplete block design with three replications was used for evaluation; each plot consisted of two 5 m long rows spaced 0.75 m apart with 0.40 m spacing between plants within rows in each trial. Three seeds were planted per hill, which was later thinned to two per stand about 2 weeks after planting, for a final plant population density of 66,000 plants/ha. Fertilizers were applied 2 weeks after planting (WAP) when necessary to bring the total available N in the soil to 30 kg ha^−1^ for the low N field and 60 kg ha^−1^ for the high N field. In addition, single superphosphate (P_2_O_5_) and muriate of potash (K_2_O) were applied to both low N and high N blocks at a rate of 60 kg ha^−1^. Additionally, 60 kg N ha ^−1^ was top-dressed at 4 WAP in the high N fields. Weeds were controlled with herbicides and/or manually.

### 4.3. Data Collection

In low and high N environments, data on the following traits were recorded and expressed as percentages: ear height (EHT = the distance from the soil level of the plant to the level of the upper ear node carrying the cob), plant height (PHT = the distance from the soil level of the plant to the first tassel branch), days to 50% anthesis (DA = the number of days from planting to the time when 50% of the plants in the plot have tassels shedding pollen), days to 50% silking (DS = the number of days from planting to the time when 50% of the plants have emerged silks), anthesis-silking interval (ASI = the difference between the two flowering dates), root lodging (RL = the number of plants that leaned more than 30° from vertical) and stalk lodging (SL = the number of plants broken at or just below the node of the upper ear). Ears per plant (EPP) = the number of ears harvested (EHARV) divided by the corresponding number of plants harvested (PHARV). Plant aspect (PASP, rated using a 1 to 9 scale) = the overall phenotypic appeal of the plants per plot as they appeared to sight, including uniformity of the plant, cobs size, disease incident, where 1 = excellent overall phenotypic appeal of the plants per plot and 9 = very poor overall appeal of the plants per plot. Ear aspect (EASP) = rated on a 1 to 9 scale, with 1 denoting large, well-filled, clean and uniform ears and 9 representing ears with undesirable characteristics. Husk cover (HUSK) = rated on 1 to 9 scale, where 1 = ears with long and tight husks that fully cover the tip and 9 = ears with exposed tips. Additionally, under low N, the data on stay-green characteristic (STGR) was measured on a scale of 1 to 9, where 1 indicates plants with less than 10% of the leaf area dead and 9 denotes plants with more than 80% of the leaf area dead. Grain weight (GWT) = measured from completely shelled ears per plot and percentage grain moisture content (GMC). Grain yield (GY, kg ha^−1^) adjusted to moisture content of 15% was derived from cob weight (field weight) and moisture content of grains per plot as follows:

(1) $$\mathrm{GY}\;(\mathrm{kg}/\mathrm{plot})=\mathrm{fw}\;(\mathrm{kg}/{\mathrm{plot}}^{-1})\;\times \;\frac{\left(100\;-\;\mathrm{mc}\right)}{\left(100\;-\;15\right)}\;\times \;\frac{10000}{\phi }\;\times \;0.8,$$

where *GY* = grain yield (kg ha^−1^), *fw*= weight of grain per plot (kg), *mc* = moisture content of grain at harvest, 10,000 m^2^ = land area ha^−1^ and *ϕ* = land area per plot.

### 4.4. Data Analysis

Combined analysis of variance (ANOVA) was carried out for agronomic traits of the four low N and five high N research environments on plot means of each trait with PROC GLM in SAS 9.3 utilizing a RANDOM statement with the TEST option [29]. In the analyses, all source of variation, except hybrids, were considered as random factors for each experiment. Repeatability for measured traits was computed for each low and high N environment as follows:

(2) $$R^{2}=\frac{\delta_{g}^{2}}{\delta_{g}^{2}+\frac{\delta_{gE}^{2}}{E}+\frac{\delta_{e}^{2}}{rE}}$$

where $\delta_{g}^{2}$ represents hybrid variance, $\delta_{gE}^{2}$ is the variance due to hybrid × environment interactions, $\delta_{e}^{2}$ is the error variance, *E* is the number of test environments and *r* is the number of replications in a test environment [30].

Regression analysis, including the parameters and graphical display of the regression line, as well as the distinction between the different periods, was performed using Excel software (Microsoft Office Suite, 2016). The gain in grain yield and other measured traits of the 54 maize hybrids over the nine-year period of development was estimated by linear regression. The genetic gain representing the regression coefficient (b value) was obtained by regressing hybrid means (dependent variable, y) of grain yield and other agronomic characteristics on the year of development (independent variable, x) in both low and high N environments. Correlation coefficients between traits were determined using SAS. Relative genetic gain per year was obtained by dividing the genetic gain (b value) by the intercept and multiplying by 100 [25]. To identify outstanding hybrids in terms of high grain yield and stability across four low and five high N environments, grain yields across replications were analyzed using the genotype main effect plus genotype × environment interaction (GGE) biplot statistical tool to partition significant hybrid × environment interaction mean squares into their components [31].

## 5. Conclusions

Annual genetic gains of 2.9% and 1.33%, with an average increase of 75.37 kg ha^−1^ year^−1^ and 55.22 kg ha^−1^ year^−1^ under low and high N conditions, respectively, were obtained. The improvement was higher during low N than high N breeding periods, indicating that considerable progress has been made in breeding for low N tolerant, early-maturing hybrids in the IITA-MIP. It may be concluded that substantial progress has been made during the past three breeding periods. Grain yield had significant correlations with all measured traits, except husk cover, ASI, days to silking and anthesis under low N. Hybrids TZdEI 314 × TZdEI 105, TZdEI 378 × TZdEI 173, ENT 12 × TZEI 48 and TZdEI 352 × TZdEI 315 were identified as outstanding and should be tested extensively in SSA and vigorously promoted for commercialization to contribute to food security.

## Figures and Tables

**Table 1:** Mean squares of grain yield and other agronomic traits of early maize hybrids of three breeding periods under low N conditions across four environments and high N conditions in five environments in Nigeria from 2017 to 2019.

Source of Variation	DF	Grain Yield, (kg/ha)	Days to Anthesis	Days to Silk	Anthesis Silking Interval	Plant Height (cm)	Ear Height (cm)	Root Lodging (%)	Stalk Lodging (%)	Husk Cover	Plant Aspect	Ear Aspect	Ear Rot	EPP	Stay-Green Characteristic
**Low N Conditions**															
Environment (E)	3	486,250 **	342.9 **	269.20 **	9.10 **	5616.10 **	3808.70 **	26.40 **	22,860.60 **	150.20 **	1.50 **	33.80 **	18,428.00 **	2.05 **	107.50 **
Block (E×Rep)	60	1,402,441 **	3.6 **	4.30 **	0.50 ns	503.10 **	262.30 **	8.50 *	15.90 ns	0.40 **	1.00 **	1.20 **	79.50 **	0.01 ns	1.10 **
Rep (E)	8	4,932,208 **	11.5 **	16.00 **	1.00 *	1721.20 **	781.20 **	43.40 **	140.30 **	0.10 ns	2.90 **	4.50 **	238.70 **	0.03 **	4.20 **
Period	2	10,464,454 **	0.7 ns	2.50 ns	1.20 ns	4699.30 **	659.80 **	3.70 ns	84.10 ns	2.80 **	5.30 **	6.80 **	9.00 ns	0.14 **	1.80 *
Hybrid (Period)	51	1,169,502 **	10.4 **	11.80 **	0.60 *	967.30 **	395.50 **	7.50 **	27.70 ns	0.70 **	0.80 **	1.30 **	55.80 **	0.01 ns	1.50 **
E×Hybrid (Period)	153	677,545 **	16 **	2.40 **	0.70 **	261.70 **	116.10 ns	6.70 ns	26.90 ns	0.50 **	0.40 *	0.60 **	34.10 **	0.01 ns	0.50 ns
E×Period	6	807,687 *	2.2 *	2.90 *	0.90 ns	210.00 ns	63.30 ns	7.00 ns	81.20 *	0.90 **	0.70 *	0.20 ns	27.50 ns	0.03 *	0.70 ns
Error	364	361,686	1	1.1	0.4	165.5	96.1	5.7	30.1	0.2	0.3	0.4	17.6	0.01	0.4
Repeatability		0.56	0.84	0.8	0.44	0.79	0.75	0.14	0.23	0.41	0.6	0.63	0.37	0.22	0.68
**High N Conditions**															
Environment (E)	4	497,614,744 **	546.36 **	122.15 **	234.61 **	42,144.65 **	12,789.53 **	4901.94 **	699.24 **	180.14 **	167.00 **	155.36 **	11,843.41 **	2.33 **	
Block (E×Rep)	75	1,120,561 **	2.05 **	2.62 **	0.89 *	345.10 **	222.56 *	48.37 ns	20.21 *	0.58 **	0.68 **	0.69 **	37.12 **	0.02 *	
Rep (E)	10	1,484,870 **	3.66 **	1.67 ns	0.89 ns	293.65 ns	463.66 **	58.80 ns	14.79 ns	1.07 **	1.13 **	0.71 ns	452.26 **	0.07 **	
Period	2	7,534,475 **	19.31 **	3.27 ns	5.04 **	6695.97 **	1607.07 **	42.45 ns	18.81 ns	2.18 **	3.49 **	3.16 **	9.23 ns	0.12 **	
Hybrid (Period)	51	2,836,305 **	18.87 **	21.29 **	1.46 **	1763.46 **	657.48 **	145.77 **	30.30 **	0.88 **	0.94 **	1.55 **	33.29 **	0.03 **	
E×Hybrid (Period)	204	1,251,778 **	2.89 **	3.27 **	1.12 **	274.62 *	175.43 ns	70.86 **	21.68 **	0.61 **	0.57 *8	0.67 **	22.76 **	0.02 **	
E×Period	8	637,817 ns	4.23 **	4.98 **	3.08 **	233.82 ns	83.93 ns	30.43 ns	10.15 ns	1.08 **	1.65 **	1.23 **	26.49 **	0.03 *	
Error	455	479,051	1.34	1.59	0.63	214.61	152.06	44.87	15.55	0.36	0.28	0.39	11.29	0.02	
Repeatability		0.59	0.86	0.85	0.47	0.86	0.77	0.51	0.35	0.33	0.38	0.56	0.30	0.24	

*, ** significant at 0.05 and 0.01 probability levels, respectively; ns = non-significant.

**Table 2:** Grain yield and other agronomic traits of eighteen early-maturing maize hybrids of three breeding periods under low N in four environments and high N in five environments in Nigeria from 2017 to 2019.

Trait	Period	Low N Conditions	High N Conditions
Grain yield (kg/ha)	2008–2010	2784.03 ± 75.11	4294.33 ± 111.79
	2011–2013	2932.54 ± 91.12	4330.10 ± 99.38
	2014–2016	3196.79 ± 66.59	4614.44 ± 116.52
Days to anthesis	2008–2010	53.19 ± 0.17	51.28 ± 0.21
	2011–2013	53.06 ± 0.23	51.29 ± 0.27
	2014–2016	53.09 ± 0.28	51.77 ± 0.35
Days to silking	2008–2010	53.99 ± 0.16	52.42 ± 0.21
	2011–2013	53.96 ± 0.23	52.52 ± 0.30
	2014–2016	53.78 ± 0.32	52.65 ± 0.37
Anthesis-silking interval	2008–2010	0.85 ± 0.07	1.33 ± 0.07
	2011–2013	0.91 ± 0.78	1.23± 0.07
	2014–2016	0.78 ± 0.06	1.05 ± 0.09
Plant height (cm)	2008–2010	157.03 ± 1.95	161.76 ± 2.42
	2011–2013	165.42 ± 2.55	170.53 ± 3.20
	2014–2016	164.18 ± 2.20	171.49 ± 2.17
Ear height (cm)	2008–2010	70.86 ± 1.46	73.96 ± 1.60
	2011–2013	74.58 ± 1.60	79.29 ± 1.91
	2014–2016	71.94 ± 1.11	76.64 ± 1.39
Root lodging %	2008–2010	0.90 ± 0.17	4.75 ± 0.89
	2011–2013	0.70 ± 0.16	5.58 ± 0.77
	2014–2016	0.99 ± 0.22	4.98 ± 0.68
Stalk lodging %	2008–2010	6.17 ± 0.34	2.89 ± 0.32
	2011–2013	6.77 ± 0.41	3.10 ± 0.40
	2014–2016	5.14 ± 0.35	2.55 ± 0.33
Husk cover	2008–2010	3.11 ± 0.06	3.41 ± 0.05
	2011–2013	3.20 ± 0.08	3.41 ± 0.06
	2014–2016	2.94 ± 0.05	3.25 ± 0.07
Plant aspect	2008–2010	4.76 ± 0.05	4.47 ± 0.06
	2011–2013	4.61 ± 0.08	4.35 ± 0.06
	2014–2016	4.39 ± 0.06	4.23 ± 0.06
Ear aspect	2008–2010	4.65 ± 0.08	3.92 ± 0.09
	2011–2013	4.34 ± 0.10	3.76 ± 0.07
	2014–2016	4.42 ± 0.07	3.69 ± 0.08
Ear rot	2008–2010	8.48 ± 0.51	6.04 ± 0.33
	2011–2013	8.51 ± 0.51	5.64 ± 0.42
	2014–2016	8.15 ± 0.58	5.81 ± 0.34
Ears per plant	2008–2010	0.80 ± 0.01	0.82 ± 0.01
	2011–2013	0.82 ± 0.01	0.86 ± 0.01
	2014–2016	0.84 ± 0.01	0.86 ± 0.01
Stay-green characteristic	2008–2010	3.31 ± 0.09	−
	2011–2013	3.22 ± 0.08	−
	2014–2016	3.14 ± 0.09	−

**Table 3:** Relative genetic gain, coefficient of determination (R^2^), slope (a) and regression coefficients (b) of grain yield and other agronomic traits of early-maturing maize hybrids during three breeding periods under low N in four environments and high N in five environments in Nigeria from 2017 to 2019.

Trait	Relative Gain (% per Year)	R^2^	a	b
**Low N**				
Grain yield (kg/ha)	2.91	0.26	2588.70	75.37 **
Days to anthesis	0.02	0.01	53.06	0.01 ns
Days to silk	−0.02	0.00	53.95	−0.01 ns
Anthesis-silking interval	−1.88	0.03	0.94	−0.02 ns
Plant height (cm)	1.07	0.17	153.89	1.64 **
Ear height (cm)	0.57	0.03	70.42	0.40 ns
Root lodging (%)	3.12	0.01	0.75	0.02 ns
Stalk lodging (%)	0.50	0.00	5.88	0.03 ns
Husk cover	−0.33	0.01	3.14	−0.01 ns
Plant aspect	−1.07	0.18	4.85	−0.05 **
Ear aspect	−1.31	0.17	4.79	−0.06 **
Ear rot	−0.80	0.01	8.74	−0.07 ns
Stay-green characteristic	−0.89	0.04	3.38	−0.03 ns
Ears/plant	1.08	0.27	0.78	0.01 **
**High N**				
Grain yield (kg/ha)	1.33	0.08	4133.30	55.12 *
Days to anthesis	0.19	0.04	50.95	0.10 ns
Days to silk	0.12	0.02	52.21	0.06 ns
Anthesis silking interval	−3.15	0.11	1.43	−0.05 *
Plant height (cm)	1.11	0.14	159.00	1.76 **
Ear height (cm)	0.84	0.05	73.51	0.62 ns
Root lodging (%)	1.87	0.00	4.66	0.09 ns
Stalk lodging (%)	−0.92	0.00	2.99	−0.03 ns
Husk cover	−0.68	0.05	3.47	−0.02 ns
Plant aspect	−0.95	0.15	4.57	−0.04 **
Ear aspect	−0.99	0.08	3.99	−0.04 *
Ear rot	−1.16	0.01	6.20	−0.07 ns
Ears/plant	0.81	0.13	0.82	0.01 **

*, ** significant at 0.05 and 0.01 probability levels, respectively; ns = non-significant.

**Table 4:** Correlation coefficients of grain yield and other agronomic traits of early-maturing maize hybrids evaluated in four low N environments (above diagonal) and five high N environments (below diagonal) in Nigeria from 2017 to 2019.

	Grain Yield, (kg/ha)	Days to Anthesis	Days to Silk	ASI	Plant Height (cm)	Ear Height (cm)	Husk Cover	Plant Aspect	Ear Aspect	Ear Rot	Ears Per Plant	Stay-Green Characteristic
**Grain yield**		0.17 ns	0.11 ns	−0.04 ns	0.47 **	0.26 *	−0.21 ns	−0.65 **	−0.61 **	−0.20 ns	0.49 **	−0.29 *
**Days to anthesis**	−0.02 ns		0.96 **	0.03 ns	0.36 **	0.41 **	−0.28 *	−0.32 *	−0.11 ns	−0.48 **	−0.17 ns	0.37 **
**Days to silk**	−0.13 ns	0.95 **		0.28 *	0.33 **	0.39 **	−0.24 ns	−0.27 ns	−0.02 ns	−0.41 **	−0.17 ns	0.35 **
**ASI**	−0.38 **	0.11 ns	0.36 **		−0.13 ns	−0.10 ns	0.17 ns	0.11 ns	0.23 ns	0.09 ns	0.04 ns	−0.02 ns
**Plant height**	0.32 **	0.27 *	0.29 *	0.02 ns		0.80 **	−0.06 ns	−0.55 **	−0.25 ns	0.00 ns	0.19 ns	0.40 **
**Ear height**	0.09 ns	0.36 **	0.42 **	0.21 ns	0.80 **		0.00 ns	−0.45 ns	−0.20 ns	−0.01 ns	0.03 ns	0.38 **
**Husk cover**	−0.07 ns	−0.32 *	−0.33 **	−0.07 ns	−0.35 **	−0.26 *		0.45 **	0.10 ns	0.34 **	−0.15 ns	0.16 ns
**Plant aspect**	−0.59 **	−0.27 *	−0.25 *	0.00 ns	−0.59 **	−0.49 **	0.60 **		0.46 **	0.26 *	−0.36 **	0.11 ns
**Ear aspect**	−0.80 **	0.02 ns	0.10 ns	0.25 *	−0.22 ns	−0.04 ns	0.11 na	0.58 **		0.28 *	−0.36 **	0.22 ns
**Ear rot**	0.04 ns	−0.42 **	−0.42 **	−0.18 ns	0.03 ns	−0.09 ns	0.37 **	0.33 *	0.20 ns		0.05 ns	0.13 ns
**EPP**	0.60 **	0.12 ns	0.07 ns	−0.19 ns	0.20 ns	0.08 ns	−0.17 ns	−0.18 ns	−0.19 ns	−0.14 ns		−0.42 **

*, ** significant at 0.05 and 0.01 probability levels, respectively; ns = non-significant.

## Data Availability

The datasets used in the present study are available in the IITA CKAN repository.

## Supplementary Materials

The following are available online at https://www.mdpi.com/article/10.3390/plants11091208/s1, Table S1: Pedigree, period and source of 54 early-maturing maize hybrids used in the study.
