# Supplementary material for: Accelerated Genetic Gains in Early-Maturing Maize Hybrids following Three Periods of Genetic Enhancement for Grain Yield under Low and High Soil-Nitrogen Environments
Source: Plants (Basel). 2022 Apr 29;11(9):1208. doi: 10.3390/plants11091208 (PMC9105051; doi:10.3390/plants11091208)
Supplement: Supplementary file 1 [file plants-11-01208-s001.zip › plants-1666049-supplementary.pdf]

Supplementary

Table S1: Pedigree, period and source of 54 early-maturing maize hybrids used in the present study.

Entry	Pedigree	Period	Source	
1	TZE-W Pop DT STR x TZEI 19	1	IITA	
2	(TZEI 63 x TZEI 87) x (TZEI 59 x TZEI 108)	1	IITA	
3	(TZEI 135 x TZEI 129) x (TZEI 16 x TZEI 157)	1	IITA	
4	TZE-Y Pop DT STR x TZEI 17	1	IITA	
5	TZEI 23 x TZEI 13	1	IITA	
6	(TZEI 63 x TZEI 59) x TZEI 87	1	IITA	
7	TZE W Pop DT STR C4 x TZEI 22	1	IITA	
8	TZEI 24 x TZEI 17	1	IITA	
9	TZEI 16 x TZEI 8	1	IITA	
10	TZE Y Pop DT STR C4 x TZEI 11	1	IITA	
11	TZEI 188 x TZEI 98	1	IITA	
12	TZEI 7 x TZEI 26	1	IITA	
13	TZEI 31 x TZEI 63	1	IITA	
14	TZEI 5 x TZEI 98	1	IITA	
15	TZEI 14 x TZEI 25	1	IITA	
16	TZEI 31 x TZEI 18	1	IITA	
17	ENT 12 x TZEI 48	1	IITA	
18	TZEI 83 x TZEI 60	1	IITA	
19	TZdEI 173 x TZdEI 352	3	IITA	
20	TZdEI 157 x TZdEI 352	3	IITA	
21	TZdEI 173 x TZdEI 280	3	IITA	
22	TZdEI 124 x TZdEI 268	3	IITA	
23	TZdEI 314 x TZdEI 105	3	IITA	
24	TZdEI 173 x TZdEI 492	3	IITA	
25	TZdEI 268 x TZdEI 131	3	IITA	
26	TZdEI 378 x TZdEI 173	3	IITA	
27	TZdEI 378 x TZdEI 98	3	IITA	
28	TZdEI 352 x TZdEI 315	3	IITA	
29	TZEI 18 x TZdEI 352	3	IITA	
30	TZdEI 479 x TZdEI 124	3	IITA	
31	TZdEI 352 x TZdEI 441	3	IITA	
32	TZdEI 17 x TZEI 17	3	IITA	
33	TZdEI 68 x TZEI 10	3	IITA	
34	TZdEI 21 x TZEI 23	3	IITA	
35	TZdEI 24 x TZEI 17	3	IITA	
36	TZdEI 21 x TZEI 10	3	IITA	

37	TZdEI 352 x TZEI 355	2	IITA	
38	TZdEI 352 x TZEI 383	2	IITA	
39	TZEI 352 x TZdEI 352	2	IITA	
40	TZEI 355 x TZdEI 425	2	IITA	
41	TZEI 326 x TZdEI 352	2	IITA	
42	TZE-W Pop DT C5 STR C5 x TZEI 63	2	IITA	
43	TZEI 326 x TZdEI 425	2	IITA	
44	TZE-Y Pop DT C5 STR C5 x ENT 13	2	IITA	
45	TZE-Y Pop DT C5 STR C5 x TZEI 129	2	IITA	
46	TZEI 495 x ENT 13	2	IITA	
47	TZEI 470 x ENT 13	2	IITA	
48	TZEI 474 x TZEI 17	2	IITA	
49	TZEI 507 x TZEI 129	2	IITA	
50	TZEI 516 x ENT 13	2	IITA	
51	TZEI 474 x TZEI 10	2	IITA	
52	TZEI 486 x TZEI 23	2	IITA	
53	TZE-Y Pop DT C5 STR C5 x TZEI 17	2	IITA	
54	TZE-Y Pop DT C5 STR C5 x TZEI 10	2	IITA	
